# Hospitalisations in patients with idiopathic pulmonary fibrosis: insights from the IPF-PRO Registry and EMPIRE Registry

**DOI:** 10.1183/23120541.00402-2025

**Published:** 2026-02-23

**Authors:** Philipp Nick, Michael Studnicka, Jakub Gregor, Petra Ovesná, Martina Koziar Vašáková, Will Simmons, Ying Li, Mary E. Strek, Peide Li, Laurie D. Snyder, Amy L. Olson, Hyun J. Kim, Robert J. Kaner

**Affiliations:** 1 Paracelsus Medical University, Salzburg, Austria; 2 Faculty of Medicine, Masaryk University, Brno, Czech Republic; 3 Clinic of Respiratory Medicine, 1st Faculty of Medicine, Charles University and Thomayer University Hospital, Prague, Czech Republic; 4 Weill Cornell Medicine, New York, NY, USA; 5 Section of Pulmonary and Critical Care Medicine, University of Chicago, Chicago, IL, USA; 6 Boehringer Ingelheim Pharmaceuticals, Ridgefield, CT, USA; 7 Duke Clinical Research Institute, Durham, NC, USA; 8 Duke University Medical Center, Durham, NC, USA; 9 Division of Pulmonary, Allergy, Critical Care, and Sleep Medicine, University of Minnesota, Minneapolis, MN, USA

## Abstract

**Background:**

We used data from the European MultiPartner IPF Registry (EMPIRE) and the US Idiopathic Pulmonary Fibrosis-PRospective Outcomes (IPF-PRO) Registry to examine the frequency of hospitalisation, risk factors associated with hospitalisation, and whether hospitalisation affected 5-year mortality in patients with idiopathic pulmonary fibrosis (IPF).

**Methods:**

Data from January 2015 to September 2022 in EMPIRE and from June 2014 to December 2023 in the IPF-PRO Registry were analysed. Rates of hospitalisation and death were estimated using the Kaplan–Meier method. Associations between patient characteristics at enrolment and time to hospitalisation were assessed using Cox regression.

**Results:**

The EMPIRE and IPF-PRO Registry cohorts comprised 2989 and 1001 patients, respectively. Median follow-up was 36.5 months in EMPIRE and 60 months in the IPF-PRO Registry. Overall, 20.3% of patients in EMPIRE and 70.3% (28.2% over 36 months) in the IPF-PRO Registry had one or more hospitalisation during follow-up. In both registries, lower percentage predicted diffusing capacity of the lungs for carbon monoxide and use of supplemental oxygen at enrolment were associated with an increased risk of hospitalisation in multivariable models. 5-year mortality did not differ between patients who were and were not hospitalised in EMPIRE (54.3% and 53.7%, respectively) or in the IPF-PRO Registry (51.7% and 46.1%, respectively).

**Conclusions:**

Data from EMPIRE and the IPF-PRO Registry demonstrate the high risk of hospitalisations and mortality among patients with IPF and suggest there may be differences across countries in risk of hospitalisation. Variability in data collection, healthcare systems and clinical practices should be considered when interpreting differences between countries.

## Shareable abstract

**Data from large European and US registries demonstrate the high risk of hospitalisations and mortality in patients with idiopathic pulmonary fibrosis** https://bit.ly/45q9go0

## Introduction

Idiopathic pulmonary fibrosis (IPF) is a chronic progressive interstitial lung disease [1]. Progression of IPF is associated with deterioration in lung function, worsening symptoms and high mortality [1]. Antifibrotic therapies (nintedanib and pirfenidone) slow the decline in lung function in patients with IPF, but the disease continues to progress [2, 3]. Data from real-world studies suggest a median survival of 4–5 years from the time that IPF is diagnosed [4, 5]. Hospitalisations are frequent in patients with IPF and are associated with poor outcomes, particularly among patients who receive mechanical ventilation [6–11].

Patient registries provide an opportunity to improve understanding of the clinical course, impact and management of IPF in the real world [12]. Compared with clinical trials, registries include broader populations of patients and collect data over longer periods of time. In this analysis, we used data from two large registries of patients with IPF in different healthcare systems, the European MultiPartner IPF Registry (EMPIRE) and the US Idiopathic Pulmonary Fibrosis-PRospective Outcomes (IPF-PRO) Registry, to examine the frequency of hospitalisation, risk factors associated with hospitalisation, and mortality in patients with IPF managed in clinical practice.

## Methods

EMPIRE is a prospective European registry of patients with IPF (https://empire.registry.cz/index-en.php). Initiated in the Czech Republic in 2012, the registry was expanded to cover other Central and Eastern European countries from 2015, and now involves pneumological centres in 11 countries. Patients aged ≥18 years with a diagnosis of IPF according to the 2011 international guidelines [13] were eligible to participate. Follow-up visits take place every 3 or 6 months, following clinical practice at each centre, until death or lung transplant. Hospitalisations were classified as having or not having a respiratory cause. The EMPIRE study protocol was approved by the relevant ethics committees prior to patient enrolment at every site. All patients provided written consent prior to entering the registry.

The IPF-PRO Registry was a prospective registry of patients with IPF conducted at 46 sites across the USA [14]. Patients aged ≥40 years with IPF that was diagnosed or confirmed at the enrolling centre in the past 6 months according to the 2011 international guidelines [13] were eligible to participate. At enrolment, retrospective data from the prior 12 months were obtained from patient records. Patients were then followed prospectively while receiving usual care, with follow-up data collected until death, lung transplant or withdrawal from the registry. Regular follow-up from a call centre ascertained vital status and interactions with the healthcare system. Hospitalisations were classified as having or not having a respiratory cause. The study was approved by the Duke University institutional review board (Pro00046131). The protocol was approved by the relevant institutional review boards and/or local independent ethics committees prior to patient enrolment at every site. All patients provided written consent prior to entering the registry.

### Data extraction

EMPIRE enrolled patients from 2012 to September 2022. The IPF-PRO Registry enrolled patients from June 2014 to October 2018. To better align data from the two registries, only data from patients enrolled in EMPIRE after 2015 and within 6 months of being diagnosed with IPF were included in this analysis. Data from January 2015 to September 2022 were extracted from the EMPIRE database and analysed at Masaryk University, Czech Republic. Data from June 2014 to December 2023 were extracted from the IPF-PRO Registry database and analysed by Boehringer Ingelheim Pharmaceuticals (Ridgefield, CT, USA).

### Analyses

Patient characteristics at enrolment in EMPIRE and the IPF-PRO Registry are presented separately. Continuous variables are presented as median (interquartile range) and categorical variables as frequency (proportion). Estimates of hospitalisation and death were calculated using the Kaplan–Meier method. Associations between patient characteristics at enrolment and time to hospitalisation were assessed using Cox regression models. The following patient characteristics, all measured at enrolment, were included as covariates in the models: age, sex, race, body mass index, smoking status (current, former, never), forced vital capacity (FVC) % predicted, diffusing capacity of the lung for carbon monoxide (*D*_LCO_) % predicted, IPF diagnostic criterion according to the 2011 American Thoracic Society/European Respiratory Society/Japanese Respiratory Society/Latin American Thoracic Association guideline (definite IPF, possible/probable IPF, or not IPF), supplemental oxygen use, and history of coronary artery disease, chronic heart failure, pulmonary hypertension, emphysema or obstructive sleep apnoea. Insurance (private insurance, no private insurance) and hospitalisation in the 12 months prior to enrolment (yes, no) were also included as covariates in analyses of data from the IPF-PRO Registry. Associations between these covariates and time to hospitalisation were assessed using univariable models, multivariable models and parsimonious models. The univariable models included each covariate individually. The multivariable models included all the covariates jointly. To develop a parsimonious list of covariates, covariates in the multivariable model were removed if they did not meet an α-to-stay criterion (or p-value) of 0.05 using stepwise backwards selection. Missing data were handled using multiple imputation, assuming that the data were missing at random with an arbitrary missing pattern. 5-year mortality in EMPIRE and the IPF-PRO Registry was compared using Kaplan–Meier survival curves and the log-rank test.

## Results

### Patients

4733 patients were enrolled in EMPIRE. Of these, 1744 patients were enrolled before 2015 and/or >6 months after diagnosis of IPF and were excluded, resulting in an analysis cohort of 2989 patients. Most patients in the EMPIRE analysis cohort came from the Czech Republic (n=1126) or Turkey (n=715) (table 1). 1002 US patients were enrolled in the IPF-PRO Registry. One patient who was in hospital when enrolled and died during that hospitalisation was excluded from the analysis cohort.

**TABLE 1:** Patient demographics and clinical characteristics in the European MultiPartner IPF Registry (EMPIRE) and the US Idiopathic Pulmonary Fibrosis-PRospective Outcomes (IPF-PRO) Registry.

	EMPIRE										IPF-PRO Registry
	Austria	Bulgaria	Czech Republic	Croatia	Hungary	Israel	Poland	Serbia	Slovakia	Turkey	USA
**Patients^#^**	77	20	1126	100	237	126	346	81	156	715	1001
**Male**	61 (79.2)	13 (65.0)	838 (74.4)	75 (75.0)	149 (62.9)	89 (70.6)	253 (73.1)	52 (64.2)	102 (65.4)	544 (76.1)	747 (74.6)
**Age years**	75 (70–81)	72 (68–76)	72 (66–77)	71 (65–75)	70 (65–75)	68 (63–75)	70 (65–77)	67 (61–73)	68 (64–74)	69 (63–74)	71 (66–75)
**BMI kg·m^−2^**	26.2 (23.9–28.3)	28.5 (25.1–31.5)	28.7 (26.1–32.0)	27.2 (24.6–30.5)	27.5 (24.9–30.2)	27.4 (24.8–30.8)	28.1 (25.6–30.7)	26.2 (24.0–28.6)	28.3 (25.8–31.2)	27.7 (25.1–30.8)	28.9 (25.9–32.4)
**White**	58 (100)	19 (100)	1122 (99.9)	100 (100)	235 (99.6)	126 (100)	346 (100)	80 (100)	155 (100)	714 (100)	931 (94.9)
**Smoking status**											
Never	22 (28.6)	10 (50.0)	492 (43.7)	13 (13.0)	98 (43.0)	55 (43.7)	81 (23.6)	39 (48.8)	85 (54.8)	216 (30.2)	331 (33.1)
Former	51 (66.2)	10 (50.0)	611 (54.3)	85 (85.0)	122 (53.5)	69 (54.8)	250 (72.9)	37 (46.3)	68 (43.9)	474 (66.3)	651 (65.1)
Current	4 (5.2)	0 (0.0)	22 (2.0)	2 (2.0)	8 (3.5)	2 (1.6)	12 (3.5)	4 (5.0)	2 (1.3)	25 (3.5)	18 (1.8)
**Diagnostic criterion for IPF**											
Definite IPF	35 (47.3)	16 (80.0)	735 (67.8)	64 (64.0)	128 (57.4)	86 (71.7)	270 (79.6)	40 (51.9)	80 (55.2)	436 (62.5)	654 (65.6)
Possible/probable IPF	38 (51.4)	4 (20.0)	342 (31.5)	31 (31.0)	86 (38.6)	25 (20.8)	67 (19.8)	29 (37.7)	57 (39.3)	221 (31.7)	343 (34.4)
Not IPF	1 (1.4)	0 (0.0)	7 (0.6)	5 (5.0)	9 (4.0)	9 (7.5)	2 (0.6)	8 (10.4)	8 (5.5)	41 (5.9)	0 (0.0)

Data are presented as n, n (% of patients with data available), or median (interquartile range). BMI: body mass index; IPF: idiopathic pulmonary fibrosis. Not all patients provided data on all variables. The characteristics of patients from Macedonia are not shown, as n=5. ^#^: total number of patients in the cohort.

### Patient characteristics at enrolment

**FIGURE 1:**
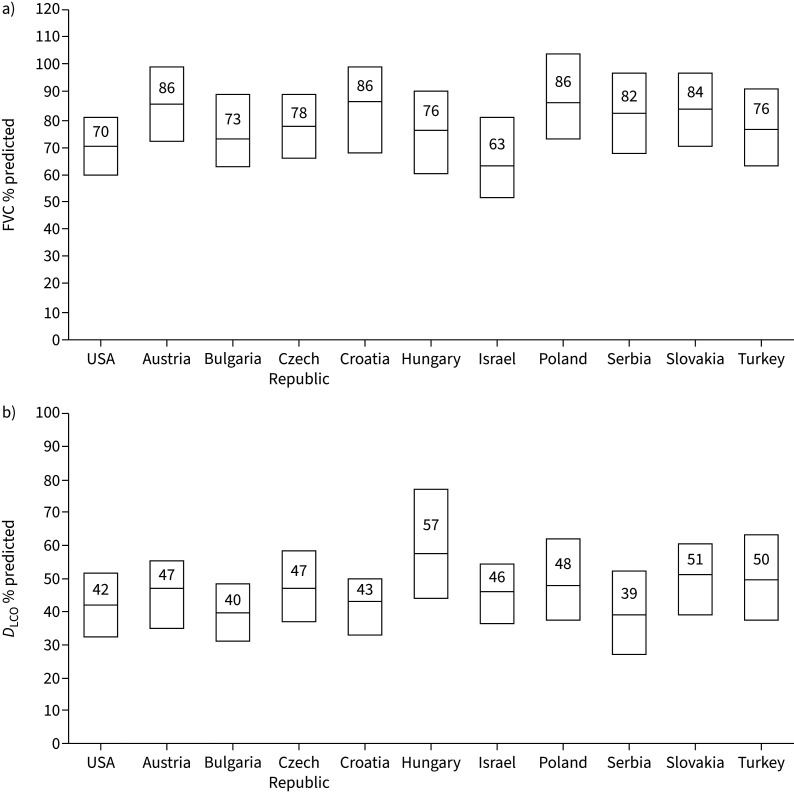
Lung function at enrolment among patients enrolled in the European MultiPartner IPF Registry (EMPIRE) and the US Idiopathic Pulmonary Fibrosis-PRospective Outcomes (IPF-PRO) Registry. a) Forced vital capacity (FVC) % predicted and b) diffusing capacity of the lung for carbon monoxide (*D*_LCO_) % predicted. Data are presented as median (interquartile range).

The demographic and clinical characteristics of the patients at enrolment (excluding the five patients enrolled in Macedonia) are shown in table 1. Median age was ∼70 years. The majority of patients were male (72.9% in EMPIRE, 74.6% in the IPF-PRO Registry). The proportion of never-smokers was similar in EMIPRE (37.4%) and the IPF-PRO Registry (33.1%). The proportion of patients with definite IPF was the same in EMPIRE and in the IPF-PRO Registry (65.6%). Median FVC was 78% predicted in EMPIRE and 70% pred in the IPF-PRO Registry. Median *D*_LCO_ was 48% predicted in EMPIRE and 42% pred in the IPF-PRO Registry. Within EMPIRE, there was a wide range in median FVC % predicted and *D*_LCO_ % predicted across countries (figure 1).

**FIGURE 2:**
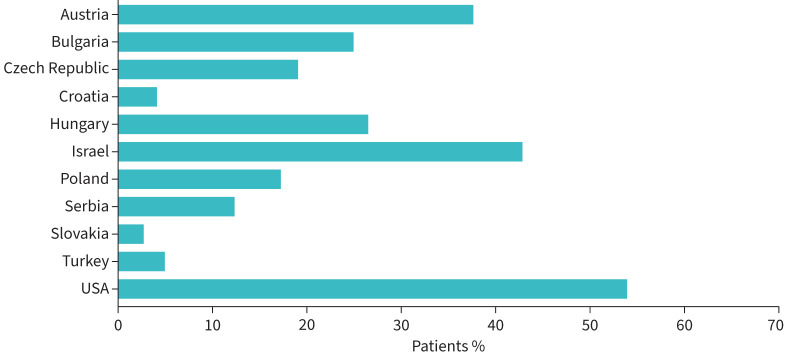
Use of antifibrotic therapy at enrolment among patients in the European MultiPartner IPF Registry (EMPIRE) and the US Idiopathic Pulmonary Fibrosis-PRospective Outcomes (IPF-PRO) Registry. Data are the percentage of patients with data available. Antifibrotic therapy used by patients from Macedonia are not shown, as n=5.

**FIGURE 3:**
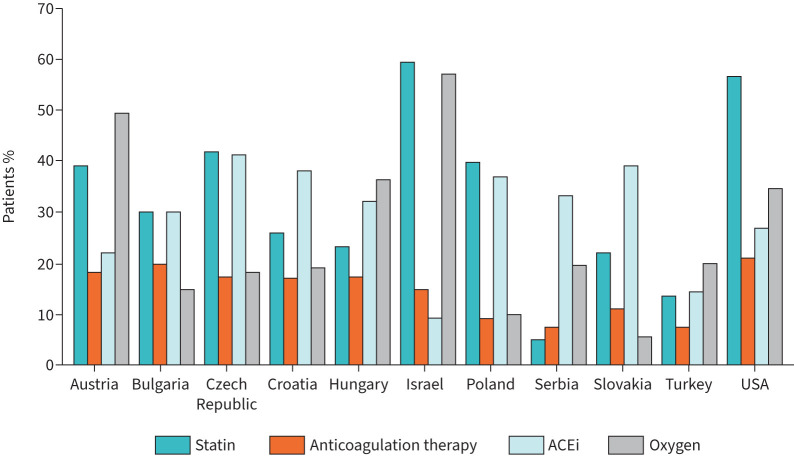
Therapies other than antifibrotic therapy used at enrolment among patients in the European MultiPartner IPF Registry (EMPIRE) and the US Idiopathic Pulmonary Fibrosis-PRospective Outcomes (IPF-PRO) Registry. Data are the percentage of patients with data available. Therapies used by patients from Macedonia are not shown, as n=5. ACEi: angiotensin-converting enzyme inhibitor.

Overall, at enrolment, 21.0% of patients from EMPIRE and 34.4% of patients from the IPF-PRO Registry were using supplemental oxygen, while 16.1% of patients from EMPIRE and 54.1% of patients from the IPF-PRO Registry were receiving antifibrotic therapy. Large differences in use of antifibrotic therapy and supplemental oxygen (as well as other medications) were also observed across the countries participating in EMPIRE (figures 2 and 3). Overall medication use in both registries was high, particularly medications related to cardiovascular disease (figure 3).

### Hospitalisations and mortality

Median follow-up was 36.5 months in EMPIRE and 60 months in the IPF-PRO Registry. Overall, 20.3% of patients in EMPIRE and 70.3% of patients in the IPF-PRO Registry had one or more hospitalisation during follow-up. Over 36 months of follow-up, 28.2% of patients in the IPF-PRO Registry had at least one hospitalisation. In univariable models, use of supplemental oxygen at enrolment was associated with an increased risk of hospitalisation, while higher FVC % predicted or *D*_LCO_ % predicted was associated with a decreased risk, in both registries (table 2). In multivariable models, use of supplemental oxygen and *D*_LCO_ % predicted at enrolment remained significantly associated with risk of hospitalisation in both registries (table 2). In parsimonious models, higher *D*_LCO_ % predicted was associated with a decreased risk, and use of supplemental oxygen with an increased risk of hospitalisation in both registries, while female sex and history of emphysema were associated with a decreased risk, and a history of pulmonary hypertension with an increased risk, in EMPIRE only (table 2).

**TABLE 2:** Associations between patient characteristics at enrolment and time to first hospitalisation in the European MultiPartner IPF Registry (EMPIRE) and the US Idiopathic Pulmonary Fibrosis-PRospective Outcomes (IPF-PRO) Registry

	Univariable models		Multivariable models		Parsimonious models	
	EMPIRE	IPF-PRO Registry	EMPIRE	IPF-PRO Registry	EMPIRE	IPF-PRO Registry
**Female**	0.82 (0.68–0.98)*	1.00 (0.84–1.18)	0.82 (0.67–1.00)*	1.06 (0.89–1.26)	0.80 (0.66–0.97)*	
**Age, per 5 years older**	1.04 (0.99–1.10)	1.09 (1.03–1.16)*	1.04 (0.98–1.09)	1.04 (0.97–1.11)		
**BMI kg·m^−2^, per 1 unit higher**	1.06 (1.00–1.13)	0.99 (0.98–1.01)	1.05 (0.99–1.12)	0.99 (0.97–1.01)		
**Current/former smoker**	1.10 (0.93–1.30)	1.27 (1.08–1.49)*	1.05 (0.87–1.25)	1.22 (1.03–1.45)*		
**Private insurance**	NA	0.85 (0.73–0.99)	NA	0.88 (0.74–1.03)	NA	0.86 (0.73–1.00)
**Diagnostic criterion of definite IPF**	1.19 (0.99–1.42)	0.93 (0.80–1.09)	1.04 (0.87–1.25)	0.93 (0.79–1.09)		
**FVC % predicted, per 10 units higher**	0.89 (0.85–0.93)*	0.91 (0.87–0.96)*	0.97 (0.92–1.02)	0.97 (0.92–1.03)		
***D*_LCO_ % predicted, per 10 units higher**	0.84 (0.80–0.89)*	0.84 (0.80–0.89)*	0.89 (0.83–0.94)*	0.91 (0.85–0.98)*	0.87 (0.82–0.93)*	0.88 (0.83–0.93)*
**Supplemental oxygen use**	2.02 (1.71–2.39)*	1.78 (1.48–2.15)*	1.73 (1.44–2.08)*	1.41 (1.10–1.80)*	1.81 (1.52–2.16)*	1.52 (1.24–1.86)*
**History of CAD or CHF**	1.34 (1.12–1.62)*	1.22 (1.05–1.43)*	1.24 (1.02–1.50)*	1.16 (0.98–1.37)		
**History of pulmonary hypertension**	1.40 (1.07–1.84)*	1.10 (0.82–1.48)	1.18 (0.89–1.57)	0.84 (0.61–1.15)	1.26 (1.05–1.52)*	
**History of emphysema**	0.82 (0.63–1.08)	1.05 (0.84–1.32)	0.63 (0.47–0.84)*	0.91 (0.72–1.15)	0.62 (0.47–0.82)	
**History of obstructive sleep apnoea**	1.41 (0.79–2.50)	1.02 (0.86–1.20)	1.09 (0.61–1.96)	1.01 (0.85–1.21)		
**Hospitalisation in the 12 months prior to enrolment**	NA	1.30 (1.09–1.55)*	NA	1.12 (0.92–1.36)	NA	1.13 (0.94–1.36)

Data are presented as hazard ratio (95% CI). BMI: body mass index; IPF: idiopathic pulmonary fibrosis; FVC: forced vital capacity; *D*_LCO_: diffusing capacity of the lung for carbon monoxide; CAD: coronary artery disease; CHF: chronic heart failure; NA: not assessed. *: p<0.05.

5-year mortality was estimated to be 54.2% in EMPIRE and 50.1% in the IPF-PRO Registry. In EMPIRE, 5-year mortality was 54.3% and 53.7% among patients who were and were not hospitalised, respectively. In the IPF-PRO Registry, 5-year mortality was 51.7% and 46.1% among patients who were and were not hospitalised, respectively.

## Discussion

These analyses of data from two registries provide further evidence that patients with IPF are at high risk of hospitalisation and mortality. This is the largest study assessing hospitalisation and predictors of hospitalisation in patients with IPF in a registry setting. We observed large differences in patient characteristics, comorbidities and medication use between patients enrolled in EMPIRE and the IPF-PRO Registry, and between the countries participating in EMPIRE. Variability in the methodology used to collect the data is likely to partly explain the observed differences in comorbidities, medications and other variables across the participating countries. Differences in medication use also reflect variability in local guidelines, clinical practice, regulatory systems and reimbursement.

A difference was observed between EMPIRE and the IPF-PRO Registry in the proportion of patients who were hospitalised (20% and 28% over 36 months of follow-up, respectively). This probably reflects differences in healthcare systems and clinical practice, cultural differences, the greater disease severity of the patients in the IPF-PRO Registry (as shown by their lower FVC and *D*_LCO_ and greater use of supplemental oxygen), as well as differences in the ascertainment of hospitalisation. It should be noted that the follow-up period in EMPIRE was much shorter than in the IPF-PRO Registry and that the IPF-PRO Registry used a call centre to obtain more data on hospitalisations in addition to the data entered at the sites. However, the EMPIRE hospitalisation rate more closely matches two analyses of insurance claims data from the USA, based on data from 1136 and 1735 patients with IPF, in which the rate of hospitalisation over 1 year was ∼38% [15, 16].

In multivariable models, use of supplemental oxygen and lower *D*_LCO_ % predicted at enrolment were significantly associated with the risk of hospitalisation in both registries. The increase in the risk of hospitalisation in patients receiving supplemental oxygen was stark (81% and 52% increases in EMPIRE and the IPF-PRO Registry, respectively). Previous studies have also shown that use of oxygen and/or lower *D*_LCO_ % predicted are associated with an increased risk of hospitalisation, and an increased risk of mortality, in patients with IPF [11, 17–20]. Both low *D*_LCO_ % predicted and supplemental oxygen use may be regarded as indicators of severe disease in patients with IPF.

Mortality rates over 5 years were ∼50% in both the EMPIRE and IPF-PRO Registry cohorts. Similarly, a meta-analysis of data from 63 307 patients with IPF from 20 countries demonstrated a cumulative 5-year survival rate of 45.6% [21]. In our analysis, mortality rates over 5 years were similar irrespective of hospitalisation. This may reflect the long time frame over which mortality was assessed. Prior studies have found that patients with IPF who are hospitalised are at high risk of mortality during or in the few months following the hospitalisation [6, 7, 9–11]. For an individual patient with IPF, the course of the disease is difficult to predict. Discussions around prognosis should be empathetic and take account of risk factors for mortality, as well as the individual patient's perceptions and concerns [22, 23].

Strengths of our analysis include the use of two large real-world cohorts of patients with IPF across 12 countries. Limitations include missing data on the variables assessed, including antifibrotic drug use, and that variables at enrolment or over time that were not evaluated may have influenced the risk of hospitalisation and survival. Associations between most comorbidities and the risk of hospitalisation could not be assessed due to differences in the methodologies used to ascertain the comorbidities across the enrolling centres. The methods used for data collection in EMPIRE and the IPF-PRO Registry differed and, as such, direct comparisons of data from these registries should be made with caution. The patients enrolled into EMPIRE and the IPF-PRO Registry may not be representative of the general population of patients with IPF in the countries included.

### Conclusions

Data from EMPIRE and the IPF-PRO Registry demonstrate the high risk of hospitalisations and mortality among patients with IPF and suggest that there may be differences across countries in the risk of hospitalisation. Variability in data collection, healthcare systems and clinical practice, as well as cultural differences, should be considered when interpreting differences between countries. Further data are needed on how acute exacerbations and other causes of hospitalisation in patients with IPF should be treated to improve outcomes.

## Data Availability

The datasets analysed during the current study are not publicly available, but are available from the corresponding author on reasonable request.
